# Identification of bone mineral density associated genes with shared genetic architectures across multiple tissues: Functional insights for *EPDR1*, *PKDCC*, and *SPTBN1*

**DOI:** 10.1371/journal.pone.0300535

**Published:** 2024-04-29

**Authors:** Jongyun Jung, Qing Wu

**Affiliations:** Department of Biomedical Informatics, College of Medicine, The Ohio State University, Columbus, Ohio, United States of America; University of Illinois at Urbana-Champaign, UNITED STATES

## Abstract

Recent studies suggest a shared genetic architecture between muscle and bone, yet the underlying molecular mechanisms remain elusive. This study aims to identify the functionally annotated genes with shared genetic architecture between muscle and bone using the most up-to-date genome-wide association study (GWAS) summary statistics from bone mineral density (BMD) and fracture-related genetic variants. We employed an advanced statistical functional mapping method to investigate shared genetic architecture between muscle and bone, focusing on genes highly expressed in muscle tissue. Our analysis identified three genes, *EPDR1*, *PKDCC*, and *SPTBN1*, which are highly expressed in muscle tissue and previously unlinked to bone metabolism. About 90% and 85% of filtered Single-Nucleotide Polymorphisms were in the intronic and intergenic regions for the threshold at *P*≤5×10^−8^ and *P*≤5×10^−100^, respectively. *EPDR1* was highly expressed in multiple tissues, including muscles, adrenal glands, blood vessels, and the thyroid. *SPTBN1* was highly expressed in all 30 tissue types except blood, while *PKDCC* was highly expressed in all 30 tissue types except the brain, pancreas, and skin. Our study provides a framework for using GWAS findings to highlight functional evidence of crosstalk between multiple tissues based on shared genetic architecture between muscle and bone. Further research should focus on functional validation, multi-omics data integration, gene-environment interactions, and clinical relevance in musculoskeletal disorders.

## Introd uction

Osteoporosis, a disease characterized by low bone mineral density (BMD), decreased bone strength, and increased fracture risk, poses a significant challenge for the aging population [1,2]. Concurrent muscle loss exacerbates this issue, further contributing to weakened bone strength and heightened susceptibility to osteoporotic fractures [3,4]. Together, osteoporosis and muscle loss represent significant risk factors for fractures in older adults [5,6].

Adequately developed muscles protect osteoporosis patients from fractures by reducing the likelihood of falls and shielding the body from osteoporotic fractures [7]. However, aging is commonly associated with the loss of skeletal muscle, which, in turn, can lead to declines in physical functioning among older adults [5,6]. Despite numerous genetic studies investigating BMD or fracture-related outcomes, the genetic link between bone and muscle remains underexplored. The exact biological pathway through which bone influences muscle is still unknown [3,8,9].

Over the past decade, genome-wide association studies (GWAS) have identified multiple genetic variants linked to BMD or fractures [10,11]. A recent study of 426,824 UK Biobank participants discovered 518 loci associated with ultrasound-derived heel BMD [10]. However, GWAS has limited success in pinpointing causal genes, with the majority of findings located in non-coding or intergenic regions [12–14]. Functional mapping is commonly used in translational research to select and prioritize genetic variants likely to be functional and quantify the strength of evidence in the GWAS [15]. In this study, we employ FUMA, a comprehensive functional mapping method that utilizes 18 biological data repositories and tools to annotate and prioritize GWAS findings [16].

Our study aims to investigate the functional consequences of BMD or fracture-related GWAS findings at the muscle tissue level, providing novel insights into the genetic architecture connecting muscle and bone. We hypothesize that identifying BMD or fracture-related GWAS findings associated with muscle loss will shed light on the genetic interplay between these two tissues and contribute to a deeper understanding of their relationship in the context of osteoporosis. Genetic variants underlying bone and muscle, identified using GWAS and statistical functional mapping methods, will provide a novel insight into targets to stratify patients for risk and develop new therapeutic strategies.

## Materials and methods

### Ethics statement

Our study does not require IRB approval since it analyzes the publicly available GWAS summary statistics data. The data available in the GWAS summary statistics to the public are not individually identifiable; therefore, the analysis would not involve human subjects.

## Genome-wide association study (GWAS) summary dataset and pre-processing

We utilized the comprehensive GWAS dataset from Morris et al. [10], which investigated genetic variants of bone mineral density (BMD) estimated by heel quantitative ultrasound (eBMD) in 426,824 UK Biobank participants. The study identified 518 genome-wide BMD-significant loci and 13 bone fracture loci. We applied two P-value thresholds (*P*≤5×10^−8^ and *P*≤5×10^−100^) to filter significant SNPs from the GWAS summary dataset. Using different P-value thresholds, we examined whether bone and muscle tissues shared different genetic architectures. A P-value threshold of *P*≤5×10^−8^ is widely used to identify associations between common genetic variants and traits. A more stringent P-value threshold of *P*≤5×10^−100^ was utilized to investigate whether rare variants contribute to the shared genetic architecture between bone and muscle. The GWAS summary statistics (UK Biobank eBMD and Fracture GWAS Data Release 2018) were obtained from the GEnetic Factor for Osteoporosis (GEFOS) consortium website [17]. Data pre-processing was performed using R version 3.6.1 software (The R Foundation) [18].

## Functional mapping with FUMA

We employed FUMA [16], a functional mapping and annotation tool, to prioritize the most likely causal single-nucleotide polymorphisms (SNPs) and genes using information from 18 biological data repositories and tools. FUMA’s core functions, SNP2GENE and GENE2FUNC processes, were used to annotate SNPs with biological functionality, map them to genes based on positional, expression quantitative trait loci, and chromatin interaction information, and gain insight into the putative biological mechanisms of prioritized genes.

## Characterization of genomic risk loci and gene annotation

Independent and significant SNPs were identified using the 1000 Genomes Project reference panel (*r*^2^<0.6). Independent lead SNPs were defined based on an *r*^2^<0.1. Genomic loci of interest were grouped if linkage disequilibrium blocks of independent and significant SNPs were closely located (<250 kb). Gene annotation was performed using ANNOVAR [19] and Ensemble genes (build 85).

## Gene-based and gene-set enrichment analysis

Gene-based GWAS analysis was conducted with MAGMA 1.6 [20] using default settings implemented in FUMA [16]. The gene-based P-values were employed for gene-set enrichment analyses, where gene expression values for 53 specific tissue types were obtained from Genotype-Tissue Expression (GTEx) [16]. Gene expression values were normalized using the log2 transformation reads per kilobase per million. At a significance level of *P*≤5×10^−8^ and *P*≤5×10^−100^, this corresponded to gene-level thresholds of 1.69×10^−5^ and 1.67×10^−3^, as well as SNP-level thresholds of 4.84×10^−7^ and 2.9×10^−5^, respectively. Functional annotation of GWAS results was performed using FUMA [16]. Normalized gene expression data (reads per kilobase per million, RPKM) for 53 tissue types were obtained from GTEx portal v6, yielding transcripts for 28,520 genes, with 22,146 mapped to the Entrez gene ID.

## Results

### Summary of genome-wide association study statistics

**Fig 1:**
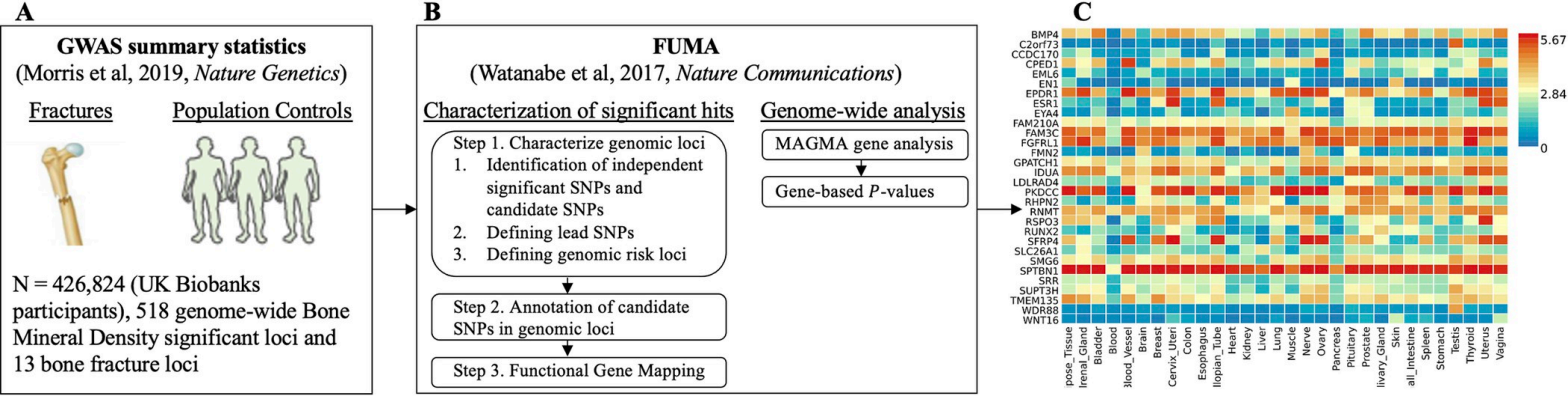
Study flow. **A**) Genome-Wide Association Study (GWAS) summary statistics of genetic association with Bone Mineral Density (BMD) or fractures for 13,753,401 Single Nucleotide Polymorphisms (SNPs) from a study estimated in 426,824 UK Biobank participants study [10]. **B**) FUMA [16] is a web-based platform utilizing information from multiple biological resources for functional annotation of GWAS results and prioritizing the most likely causal SNPs using information from 18 biological data repositories, including the Genotype-Tissue Expression (GTEx) data. Characterization of significant hits and Genome-wide analysis were conducted with FUMA. **C**) Heatmap shows the highly expressed genes in multiple tissues of GTEx data.

We analyzed previously published summary statistics from a genome-wide association study (GWAS) on bone mineral density estimated from quantitative heel ultrasounds (eBMD) [10], which included genotyping and imputed data for up to 426,824 participants in the U.K. Biobank study available from the GEnetic Factor for OSteoporosis consortium (GEFOS) website [17] (**Fig 1A**). We performed two primary analyses using FUMA: characterization of significant hits and genome-wide analysis (**Fig 1B**).

**Fig 2:**
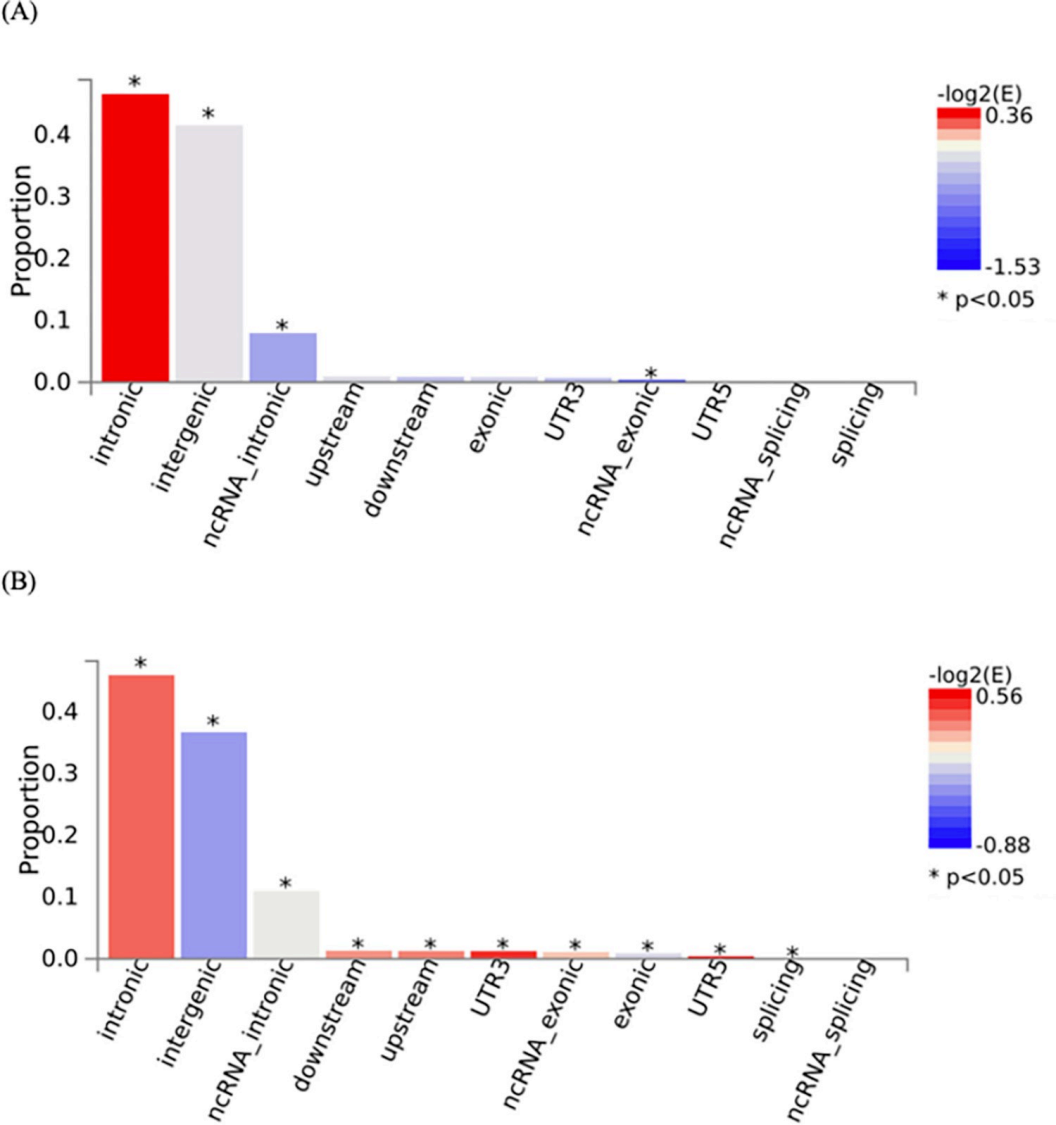
Functional consequences of Single Nucleotide Polymorphisms (SNPs) with two different thresholds. Two different P-value thresholds (A) *P*≤5×10^−8^ and (B) *P*≤5×10^−100^ were used to filter the Genome-Wide Association Study (GWAS) Summary Statistics. The P-value was computed using the chi-square statistics.

The eBMD GWAS summary statistics contained data for 13,753,401 SNPs. We filtered the summary statistics using two thresholds: *P*≤×10^−8^ and *P*≤5×10^−100^. These thresholds yielded 103,155 and 1,724 candidate GWAS-tagged SNPs, mapping to 2,955 and 30 genes, respectively (**Table 1**). Approximately 90% and 85% of filtered SNPs were in intronic and intergenic regions at *P*≤5×10^−8^ and *P*≤5×10^−100^ thresholds, respectively (**Fig 2**). The summary per genomic risk locus plot revealed that genomic loci 6:44683838–45404170 had the largest size (>700 kb), followed by 2:119014660–119634677 (>600 kb) and 2:54616729–55003484 (>400 kb) (**S1 Fig**). Over 550 SNPs were found in the genomic loci—7:120703929–121098222.

**Table 1:** The summary of Genome-Wide Association Study (GWAS) summary statistics for Single-Nucleotide Polymorphisms (SNPs) and mapped genes. Two different *P*− value thresholds were used to filter the GWAS summary statistics.

	*P*≤5×10^−8^	*P*≤5×10^−100^
Number of candidates GWAS tagged SNPs	103,155	1,724
Number of lead SNPs	2,410	40
Number of genomic risk loci	600	25
Number of mapped genes	2,955	30

**Fig 3:**
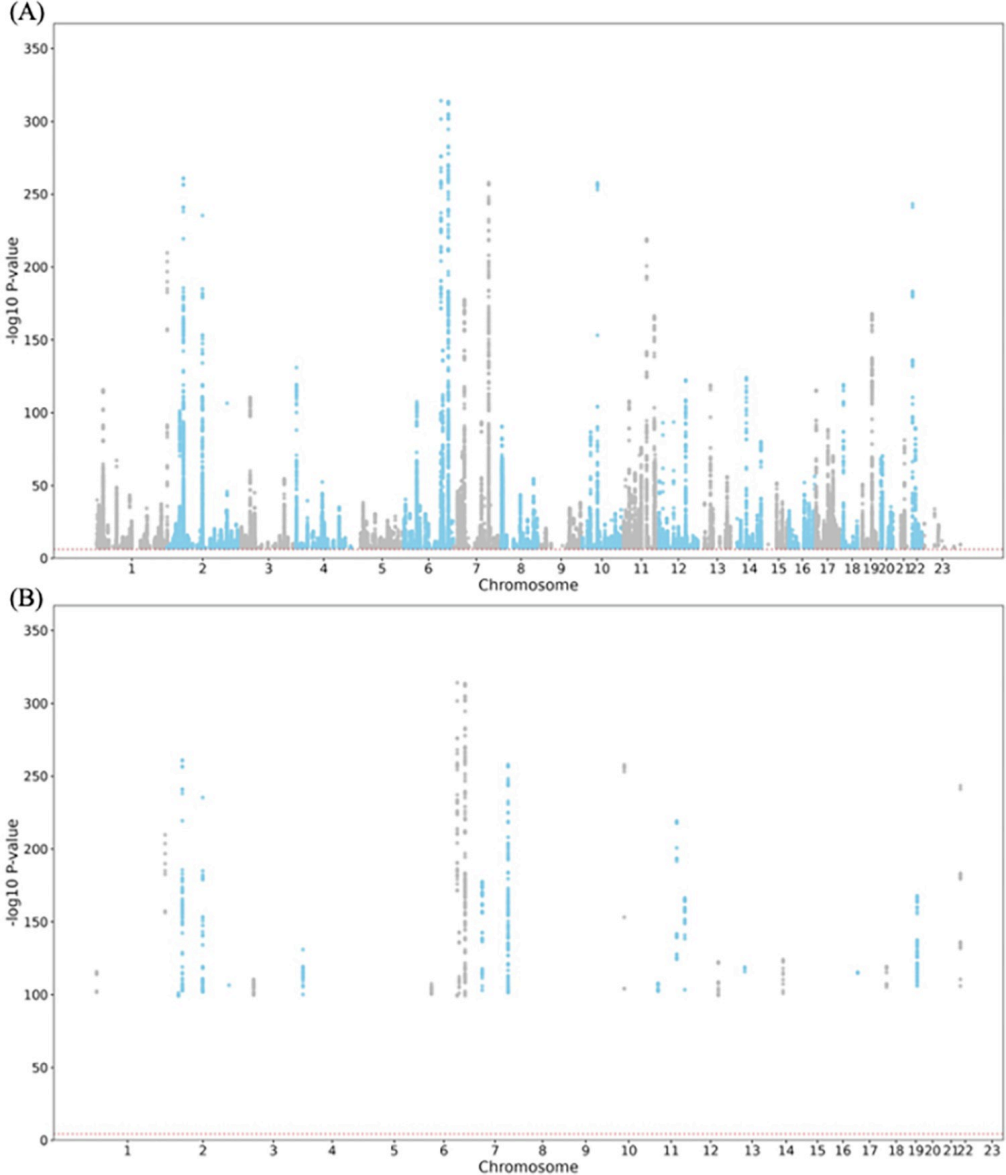
Manhattan Plot at Single Nucleotide Polymorphisms (SNPs) level. (A) *P*≤5×10^−8^ and (B) *P*≤5×10^−100^ were used to filter the Genome-Wide Association Study (GWAS) Summary Statistics. The plot shows the p-values on a log-10 scale (y-axis) by their chromosomal location (x-axis). Genome-wide significance (Red dashed line in the plot) was defined at (A) p = 0.05/103155 = 4.84×10^−7^ and (B) p = 0.05/1724 = 2.9×10^−5^, respectively.

**Fig 4:**
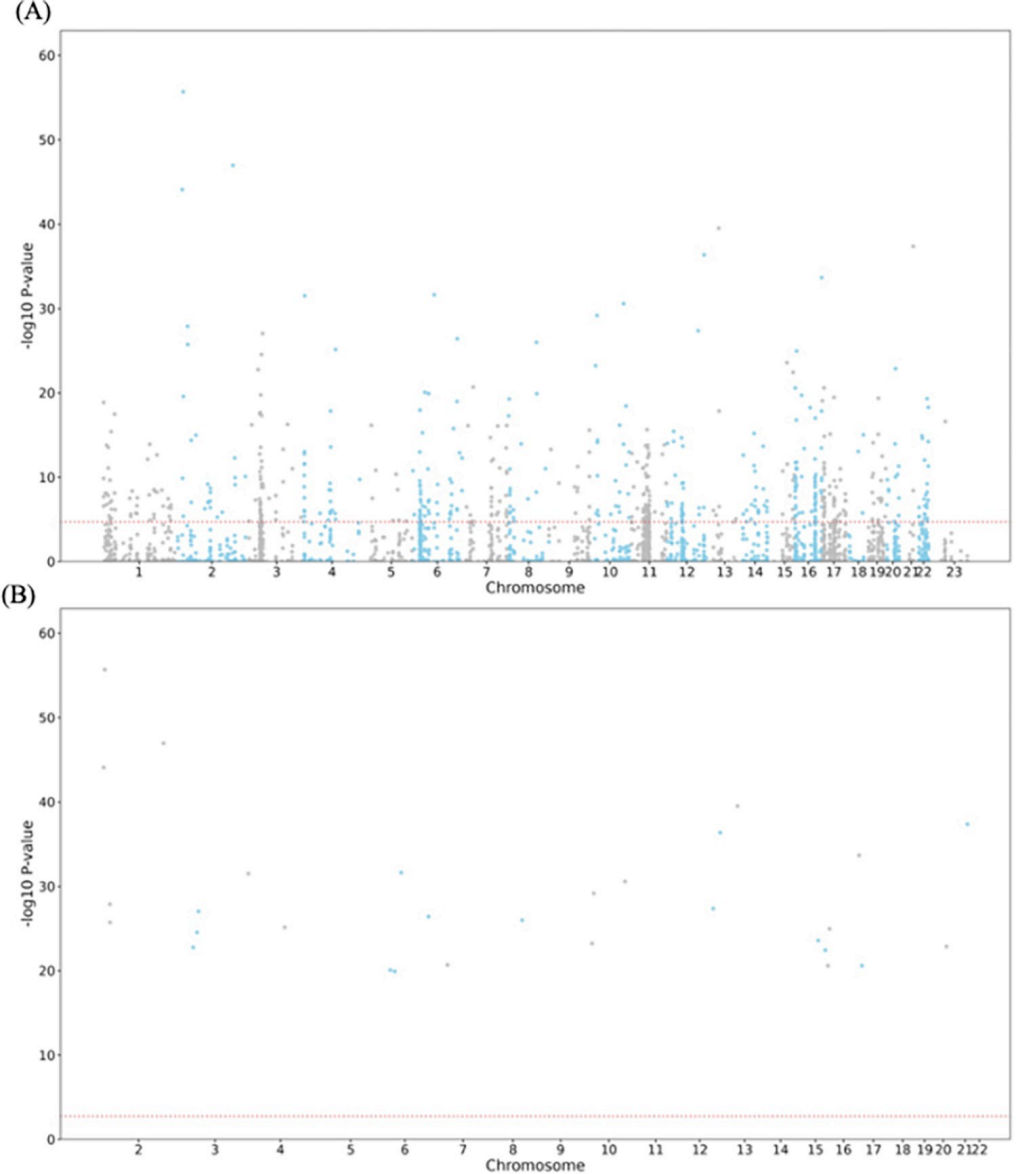
Manhattan Plot at the gene level. (A) *P*≤5×10^−8^ and (B) *P*≤5×10^−100^ were used to filter the Genome-Wide Association Study (GWAS) Summary Statistics. The plot shows the p-values on a log-10 scale (y-axis) by their chromosomal location (x-axis). Genome wide significance (Red dashed line in the plot) was defined at (A) p = 0.05/2955 = 1.69×10^−5^ and (B) p = 0.05/30 = 1.67×10^−3^, respectively.

Manhattan plots at the SNP level for both thresholds indicated that chromosomes 2, 6, 7, 10, and 22 contained the most significant SNPs (**Fig 3**). At the gene level, multiple genes were identified, including *NKX1-1*, *ZNF800*, *EML6*, *EN1*, and *LDLRAD4* (**Fig 4**).

### Gene expression analysis at the tissue level

**Fig 5:**
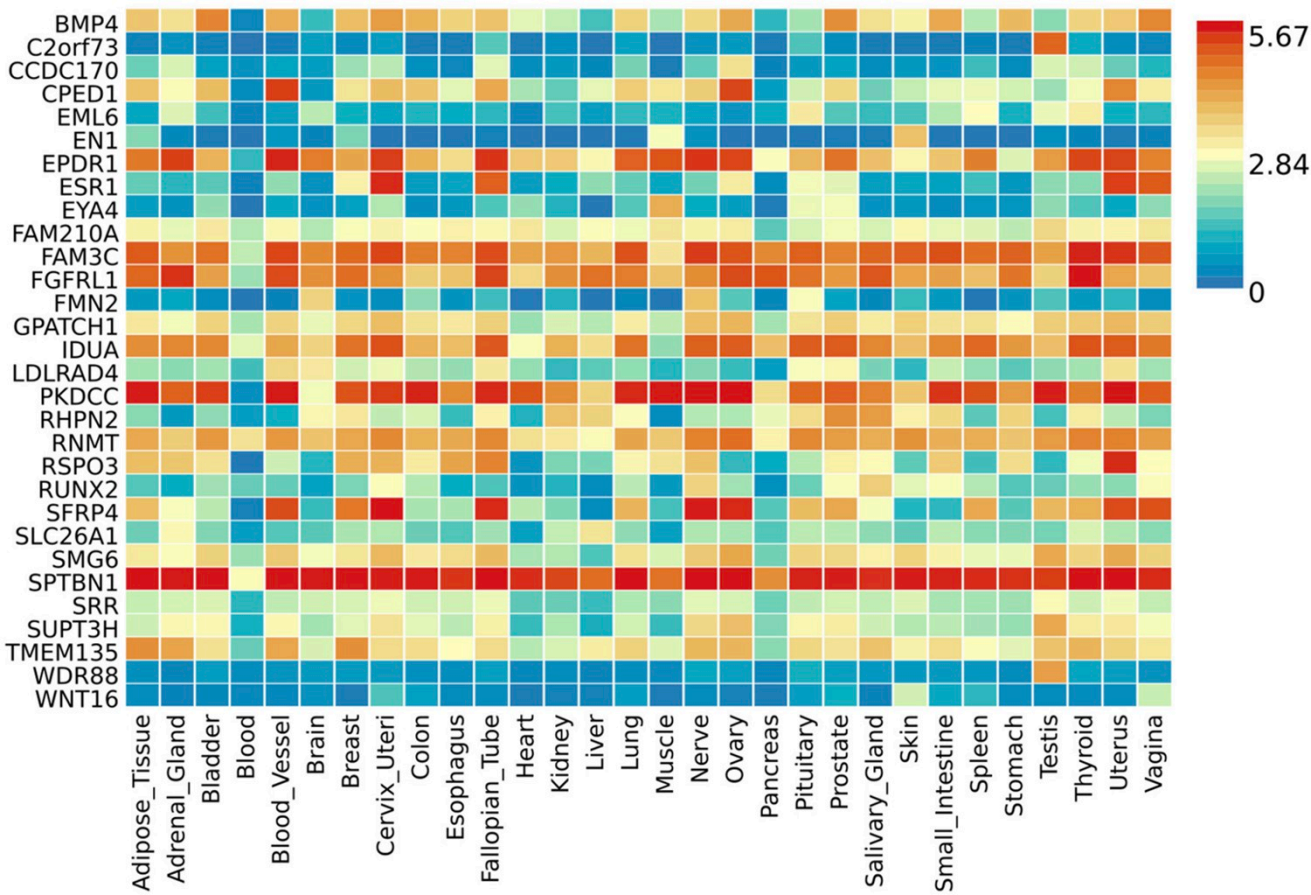
Gene expression heatmap with the Genotype-Tissue Expression v8 representing 30 general tissue types. Functional Mapping and Annotation of Genome-Wide Association Studies (FUMA GWAS) GENE2FUNC online tool was used to create a heatmap showing gene expression datasets using log2 transformed expression values from a threshold *P*≤5×10^−100^ was used to map the genes. Red cells depict higher expression compared to cells filled in blue and yellow represents expression that is not significantly different from other genes.

**Fig 6:**
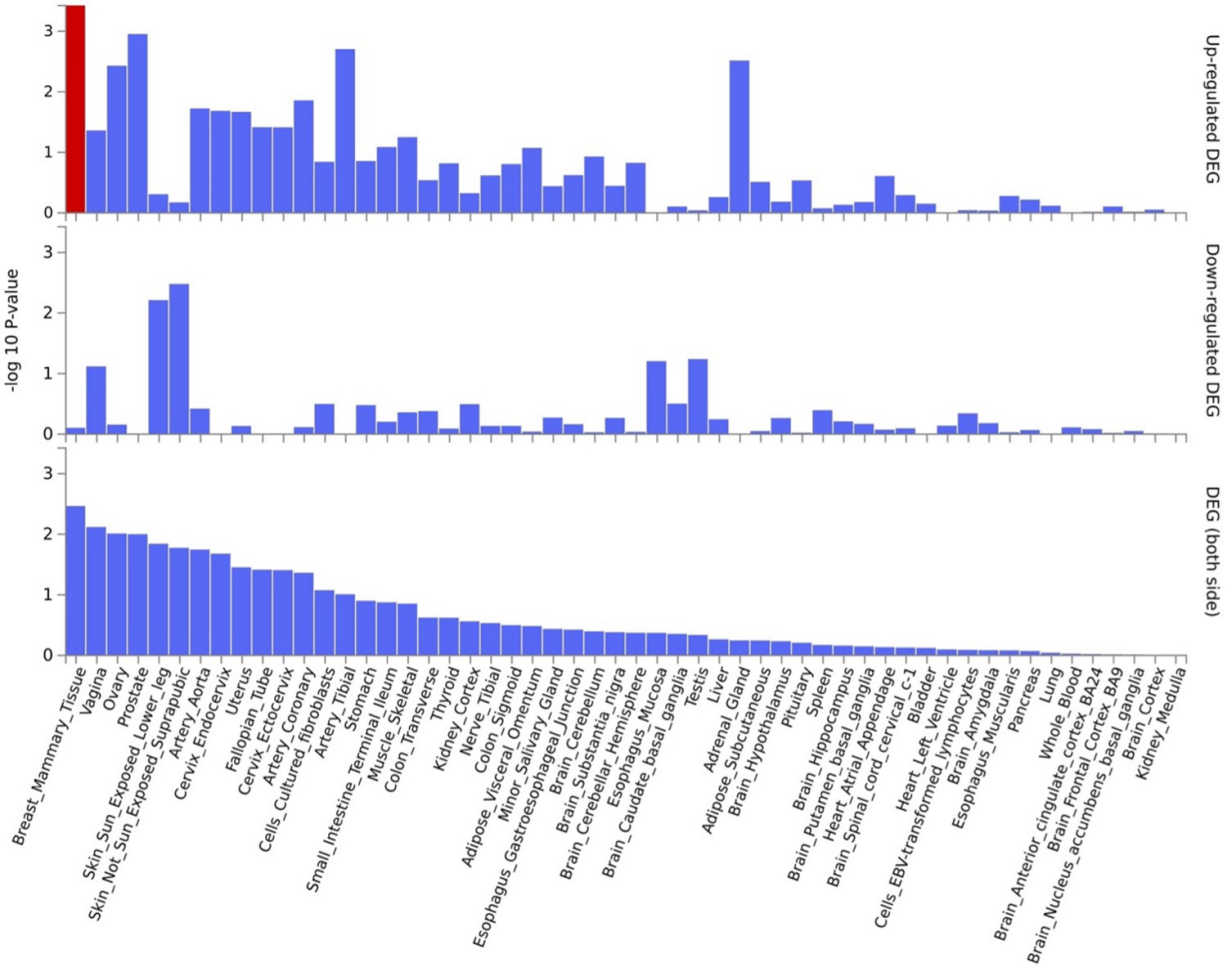
Tissue enrichment analysis using MAGMA [20]. The most enriched tissue is the mammary breast tissue. Significantly enriched DEG sets (*P*_*bon*_<0.05) are highlighted in red. A threshold *P*≤5×10^−100^ was used to map the genes.

**Fig 7:**
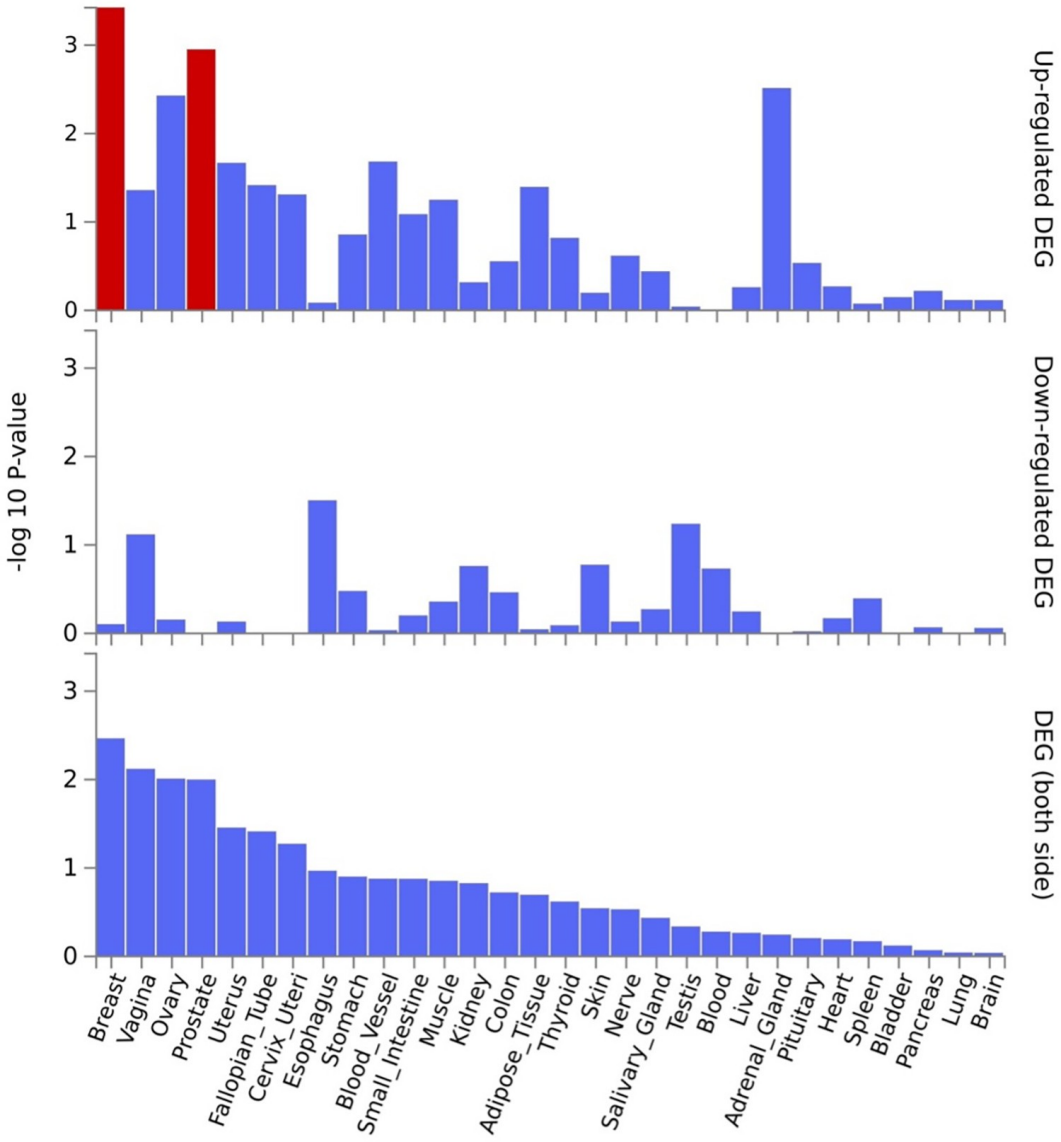
Differentially expressed genes (DEG) in 30 major tissues in the GTEx data. A threshold *P*≤5×10^−8^ was used to map the genes. Significantly expressed DEG sets (*P*_*bon*_<0.05) are highlighted in red.

*EPDR1*, *PKDCC*, and *SPTBN1* genes showed high expression levels in muscle tissue (**Fig 5**). *EPDR1* was also highly expressed in other tissues, such as the adrenal gland, blood vessel, cervix uteri, fallopian tube, nerve, ovary, thyroid, and uterus. *SPTBN1* demonstrated high expression across all 30 tissue types except blood, while *PKDCC* was highly expressed in all tissue types except the brain, pancreas, and skin. Tissue enrichment analysis using MAGMA results indicated that the most enriched tissue was the mammary breast tissue at the *P*≤5×10^−100^ threshold (**Fig 6**), and several other tissues, including fallopian tube, ovary, kidney cortex, kidney medulla, breast mammary, cervix endocervix, and adipose visceral omentum, were enriched at the *P*≤5×10^−8^ threshold (**S2 Fig**). Analysis of differentially expressed genes (DEG) across 30 major tissues using GTEx data reveals that, at the 5×10^−8^ threshold with 2,955 mapped genes, there are up-regulated DEG in breast and prostate tissues (**Fig 7**). However, when applying a more stringent threshold at the *P*≤5×10^−100^ with 30 genes, no significant DEG was observed (**S3 Fig**).

## Discussion

Our study identified three novel genes—*EPDR1*, *PKDCC*, and *SPTBN1*—highly expressed in muscle tissue using the most updated GWAS summary statistics from bone mineral density estimated from quantitative heel ultrasounds (eBMD) and fracture-related genetic variants, coupled with advanced statistical fine-mapping methods. These genes were not previously linked to both muscle and bone tissues, providing new insights into the common genetic framework. Our findings support the existence of pleiotropy between muscle and bone, as previously suggested [21].

In a prior investigation, Huang et al. [22] identified *METTL21c* as a pleiotropy gene impact on both bone and muscle. Their approach involved a bivariate genome-wide association study, which led them to pinpoint a specific genetic locus marked by the variant rs895999 (*p* = 2.3 ×10^−7^), located within the *METTL2* family of the methyltransferase superfamily. However, subsequent examination of rs895999 using the GWAS dataset published by Morris et al. [10] revealed a P-value of 0.52 for the eBMD. Consequently, this variant did not meet our selection criteria and was not subjected to functional gene annotation in our study.

Recent research highlights the significance of three identified genes (*EPDR1*, *PKDCC*, and *SPTBN1*) in diverse functions and disease contexts (**Table 2**). Prior research has demonstrated that *EPDR1* shows a high expression in the brain, muscle, heart, and extracellular fluids [23] and plays a crucial role in energy metabolism and human plasma dynamics [24]. Our study further demonstrated *EPDR1* expression in the adrenal gland, blood vessels, cervix uteri, fallopian tube, nerve, ovary, thyroid, and uterus. Although *PKDCC* has not been previously linked to bone mineral density or fracture-related outcomes, it serves multifaceted functions in intracellular signaling pathways [25]. Our study found that *PKDCC* is highly expressed in all 30 tissue types except the brain, pancreas, and skin. *SPTBN1*, a vital protein, plays a pivotal role in maintaining cellular architecture function across various tissues, including skeletal and cardiac muscle [26]. Beyond providing structural stability and participating in intracellular signaling pathways [27], *SPTBN1* plays a crucial role in bone metabolism [28]. Rao et al. [29] identified a significant role for *SPTBN1* in nonalcoholic steatohepatitis and liver cancer, while Chen et al. [30] revealed its involvement in hepatocellular carcinoma carcinogenesis. Our study broadens the comprehension of biological pathways that impact both muscle and bone, aligning with previous documentation [9,21,31], and our findings supplement earlier GWAS research [28,32].

**Table 2:** Summary of known and novel findings for three identified genes (*EPDR1*, *PKDCC*, and *SPTBN1*).

Gene	Known Evidence	Novel Findings
EPDR1	• Highly expressed in the brain and detected in other tissues, such as muscle, heart, and extracellular fluids [23]. • Plays a critical role in the context of energy metabolism and detected in human plasma [24]. • Plays a crucial role in the osteoblastogenesis [26,27].	• Highly expressed in the adrenal gland, blood vessel, cervix uteri, fallopian tube, nerve, ovary, thyroid, and uterus.
PKDCC	• Has multifaceted functions in intracellular signaling pathways [25].	• Highly expressed in all 30 tissue types except the brain, pancreas, and skin.
SPTBN1	• Plays a significant role in nonalcoholic steatohepatitis, liver cancer [29] and hepatocellular carcinoma carcinogenesis [30]. • Critical protein that maintains cellular architecture and function in various tissues, including skeletal and cardiac muscle [33]. • Provides structure stability and plays a role in intracellular signaling pathways [34]. • Plays a crucial role in bone metabolism [35].	• Highly expressed in all 30 tissue types except the blood. • Maintain the shared genetic architecture between bone and muscle.

While our study implicates *SPTBN1* in the context of osteoporosis primarily based on genome-wide association study (GWAS) data, we acknowledge existing literature supporting its involvement in bone health. The study by Calabrese et al. [36], employed a bone co-expression network to predict causal genes at bone mineral density (BMD) GWAS loci, identifying *SPTBN1* as potentially responsible for effects on chromosome 2p16.2. Furthermore, Xu et al., [37] demonstrated the role of *SPTBN1* in suppressing primary osteoporosis by influencing osteoblast proliferation, differentiation, and apoptosis through the TGFβ/Smad3 and STAT1/Cxcl9 pathways. These findings collectively suggest that *SPTBN1* plays a significant role in bone metabolism and osteoporosis pathogenesis. *EPDR1* is associated with osteoblastogenesis, the formation of osteoblasts, the bone-forming cells [26]. Studies have shown that downregulation of *EPDR1* expression inhibits osteoblastogenesis, suggesting that *EPDR1* plays a crucial role in this process [26,27]. Furthermore, genome editing experiments using CRISPR-Cas9 technology have demonstrated that alterations in *EPDR1* expression led to changes in osteoblast differentiation markers, such as alkaline phosphatase staining, further supporting its involvement in osteoblastogenesis. Overall, these findings highlight *EPDR1* as a potential key regulator of osteoblast differentiation and bone formation.

Significantly, our study reveals that three identified genes (*EPDR1*, *PKDCC*, and *SPTBN1*) show high expression not only in muscle tissue but also in various other tissues, including adipose tissue, adrenal gland, bladder, blood vessels, breast, uterus cervix, colon, fallopian tube, heart, kidney, lung, nerve, ovary, pituitary, prostate, salivary gland, small intestine, spleen, testis, thyroid, uterus, and vagina. Notably, *SPTBN1* demonstrates high expression in all tissues, excluding blood. This novel finding may be caused by the presence of tissue-specific promoters governing *SPTBN1* expression [38]. The existence of tissue-specific promoters in non-blood tissues could facilitate the activation of *SPTBN1* while maintaining its suppression in blood. Additionally, epigenetic marks may favor the activation of *SPTBN1* in specific tissues while potentially suppressing it in blood [39].

Another important finding in our analysis is that 90% and 85% of filtered SNPs were in intronic and intergenic regions for the thresholds at *P*≤5×10^−8^ and *P*≤5×10^−100^, respectively. Although GWAS have identified numerous genetic variants associated with BMD and fracture, their applicability to functional studies remains limited. Consequently, additional genetic variants might be relevant to the relationship between muscle and bone genetic architecture that were not captured in our analysis. In our tissue enrichment analysis employing both *P* ≤5 ×10^−8^ (encompassing 2955 gens) and *P* ≤5 ×10^−100^ (encompassing 30 genes) thresholds, it is important to note that muscle tissue did not emerge as significantly enriched. The stringent nature of the *P* ≤5 ×10^−100^ threshold, coupled with the identification of only 30 genes enriched in breast tissue at this low threshold, implies the highly specific and critical nature of these genes to breast-related functions. Considering the likelihood of certain genes exhibiting high specificity to particular tissues [40,41], the enrichment observed in the fallopian tube, ovary, kidney cortex, kidney medulla, breast mammary, cervix endocervix, and adipose visceral omentum at the less stringent *P* ≤5 ×10^−8^ thresholds suggests a broader set of genes associated with these tissues, distinct from those implicated in muscle tissue. This underscores the potential existence of tissue-specific genetic signatures that may play distinct roles in the genetic architecture of various tissues.

Our findings should be interpreted in the context of certain limitations. First, the GWAS summary statistics used in our study were derived from a White population; future research should validate our findings in other racial/ethnic people. Second, our analysis assumes that GWAS findings from fractures or BMD are related to muscle tissue, but this approach may not capture all relationships between muscle and bone genetic architecture. Trajanoska et al. [9] highlighted the influence of mitochondrial genetics on bones and muscles, which standard GWAS cannot reliably assess due to the sparse number of mitochondrial markers on genotyping arrays and difficulties in quantifying mitochondrial heteroplasmy. Moreover, many haplotype blocks containing GWAS SNPs do not overlap with regions of known function and remain classified as intronic or intergenic [42]. Third, the GWAS summary statistics used in our study for BMD were derived from the heel quantitative ultrasound; thus, future research is needed to validate our findings using the DXA-derived hip or spine BMD, which is typically measured in the clinic. Lastly, while our study identifies a correlation between the high expression of the identified genes in skeletal muscle and bone, we recognize the need for caution in inferring causation. We plan to investigate the perturbation of these genes specifically in skeletal muscle during conditions such as osteoporosis. This approach will provide valuable insights into the functional roles of these genes not only in bones but also in the context of skeletal muscle physiology and pathologies.

In conclusion, our study has identified three new genes—*EPDR1*, *PKDCC*, and *SPTBN1*—highly expressed in muscle tissue, providing novel insights into the shared genetic architecture between muscle and bone. Furthermore, we offer a framework for utilizing GWAS findings to emphasize functional evidence of crosstalk between multiple tissues based on other genetic architecture. Future research should explore the implications of these findings and investigate potential applications in developing therapeutic strategies targeting both muscle and bone health.

Further research directions to build on our results include: 1) integrating multi-omics data, such as transcriptomics, proteomics, and epigenomics, to uncover the molecular mechanisms linking the identified genes to muscle and bone tissues, 2) validating our novel findings in animal experiments, 3) examining associations between *EPDR1*, *PKDCC*, and *SPTBN1* expression and the development or progression of musculoskeletal disorders in longitudinal cohort studies to establish their clinical relevance, and 4) exploring novel therapeutic strategies targeting the products or regulatory elements of the identified genes to prevent or treat musculoskeletal disorders. By pursuing these research avenues, we aim to achieve a more comprehensive understanding of the shared genetic architecture between muscle and bone, ultimately benefiting the development of therapeutic interventions targeting both tissues.

## Supporting information

S1 Fig The summary per genomic risk locus. Note that genomic loci could contain more than one independent lead Single-Nucleotide Polymorphisms (SNPs).
(PDF)

S2 Fig Tissue enrichment analysis using MAGMA [20]. The most enriched tissue is the fallopian tube. Significantly enriched DEG sets (*P*_*bon*_<0.05) are highlighted in red. A threshold *P* ≤5 ×10^−8^ was used to map the genes. The horizontal dashed line indicates a Bonferroni-corrected significance threshold with 53 tissue types (0.05/53).
(PDF)

S3 Fig Differentially expressed genes (DEG) in 30 major tissues in the GTEx data. A threshold *P* ≤5 ×10^−100^ was used to map the genes.
(PDF)

## References

[1] Mesquita PN, Maia JMC, Bandeira F. Postmenopausal osteoporosis. Endocrinol Diabetes Probl-Oriented Approach. 2014;9781461486:305–21.

[2] World Health Organization. Consensus development conference: Prophylaxis and treatment of osteoporosis. Osteoporos Int. 1991;1:114–7. 1790392

[3] Burr DB. Muscle Strength, Bone Mass, and Age-Related Bone Loss. J Bone Miner Res. 1997;12:1547–51. doi: 10.1359/jbmr.1997.12.10.1547 9333114

[4] Bettis T, Kim B-J, Hamrick MW. Impact of muscle atrophy on bone metabolism and bone strength: implications for muscle-bone crosstalk with aging and disuse. Osteoporos Int. 2018;29:1713–20. doi: 10.1007/s00198-018-4570-1 29777277 PMC7861141

[5] Di Monaco M, Vallero F, Di Monaco R, Tappero R. Prevalence of sarcopenia and its association with osteoporosis in 313 older women following a hip fracture. Arch Gerontol Geriatr. 2011;52:71–4. doi: 10.1016/j.archger.2010.02.002 20207030

[6] Frisoli A, Chaves PH, Ingham SJM, Fried LP. Severe osteopenia and osteoporosis, sarcopenia, and frailty status in community-dwelling older women: Results from the Women’s Health and Aging Study (WHAS) II. Bone. 2011;48:952–7. doi: 10.1016/j.bone.2010.12.025 21195216

[7] Avin KG, Bloomfield SA, Gross TS, Warden SJ. Biomechanical Aspects of the Muscle-Bone Interaction. Curr Osteoporos Rep. 2015;13:1–8. doi: 10.1007/s11914-014-0244-x 25515697 PMC4306629

[8] Tagliaferri C, Wittrant Y, Davicco M-J, Walrand S, Coxam V. Muscle and bone, two interconnected tissues. Ageing Res Rev. 2015;21:55–70. doi: 10.1016/j.arr.2015.03.002 25804855

[9] Trajanoska K, Rivadeneira F, Kiel DP, Karasik D. Genetics of Bone and Muscle Interactions in Humans. Curr Osteoporos Rep. 2019;17:86–95. doi: 10.1007/s11914-019-00505-1 30820831 PMC6424938

[10] Morris JA, Kemp JP, Youlten SE, Laurent L, Logan JG, Chai RC, et al. An atlas of genetic influences on osteoporosis in humans and mice. Nat Genet. 2019;51:258–66. doi: 10.1038/s41588-018-0302-x 30598549 PMC6358485

[11] Kemp JP, Morris JA, Medina-Gomez C, Forgetta V, Warrington NM, Youlten SE, et al. Identification of 153 new loci associated with heel bone mineral density and functional involvement of GPC6 in osteoporosis. Nat Genet. 2017;49:1468–75. doi: 10.1038/ng.3949 28869591 PMC5621629

[12] Rocha-Braz MGM, Ferraz-de-Souza B. Genetics of osteoporosis: Searching for candidate genes for bone fragility. Arch Endocrinol Metab. 2016;60:391–401. doi: 10.1590/2359-3997000000178 27533615 PMC10118722

[13] Sabik OL, Farber CR. Using GWAS to identify novel therapeutic targets for osteoporosis. Transl Res. 2017;181:15–26. doi: 10.1016/j.trsl.2016.10.009 27837649 PMC5357198

[14] Maurano MT, Humbert R, Rynes E, Thurman RE, Haugen E, Wang H, et al. Systematic localization of common disease-associated variation in regulatory DNA. Science. 2012;337:1190–5. doi: 10.1126/science.1222794 22955828 PMC3771521

[15] Schaid DJ, Chen W, Larson NB. From genome-wide associations to candidate causal variants by statistical fine-mapping. Nat Rev Genet. 2018;19:491–504. doi: 10.1038/s41576-018-0016-z 29844615 PMC6050137

[16] Watanabe K, Taskesen E, Van Bochoven A, Posthuma D. Functional mapping and annotation of genetic associations with FUMA. Nat Commun. 2017;8:1–11. 29184056 10.1038/s41467-017-01261-5 PMC5705698

[17] GEFOS. UK Biobank eBMD and Fracture GWAS Data Release 2018 [Internet]. GEFOS. 2018 [cited 2020 Mar 5]. Available from: http://www.gefos.org/?q=content/data-release-2018

[18] Team RC. R: A language and environment for statistical computing. R Found Stat Comput Vienna Austria [Internet]. 2019;3. Available from: https://www.r-project.org/

[19] Wang K, Li M, Hakonarson H. ANNOVAR: Functional annotation of genetic variants from high-throughput sequencing data. Nucleic Acids Res. 2010;38:1–7. 20601685 10.1093/nar/gkq603 PMC2938201

[20] de Leeuw CA, Mooij JM, Heskes T, Posthuma D. MAGMA: Generalized Gene-Set Analysis of GWAS Data. Tang H, editor. PLOS Comput Biol. 2015;11:e1004219. doi: 10.1371/journal.pcbi.1004219 25885710 PMC4401657

[21] Karasik D, Kiel DP. Genetics of the Musculoskeletal System: A Pleiotropic Approach. J Bone Miner Res. 2008;23:788–802. doi: 10.1359/jbmr.080218 18269309 PMC3280426

[22] Huang J, Hsu Y-H, Mo C, Abreu E, Kiel DP, Bonewald LF, et al. METTL21C Is a Potential Pleiotropic Gene for Osteoporosis and Sarcopenia Acting Through the Modulation of the NF-κB Signaling Pathway. J Bone Miner Res. 2014;29:1531–40. 24677265 10.1002/jbmr.2200 PMC4074268

[23] Chen R, Zhang Y. EPDR1 correlates with immune cell infiltration in hepatocellular carcinoma and can be used as a prognostic biomarker. J Cell Mol Med. 2020;24:12107–18. doi: 10.1111/jcmm.15852 32935479 PMC7579695

[24] Deshmukh AS, Peijs L, Beaudry JL, Jespersen NZ, Nielsen CH, Ma T, et al. Proteomics-Based Comparative Mapping of the Secretomes of Human Brown and White Adipocytes Reveals EPDR1 as a Novel Batokine. Cell Metab. 2019;30:963–975.e7. doi: 10.1016/j.cmet.2019.10.001 31668873

[25] Sajan SA, Ganesh J, Shinde DN, Powis Z, Scarano MI, Stone J, et al. Biallelic disruption of PKDCC is associated with a skeletal disorder characterised by rhizomelic shortening of extremities and dysmorphic features. J Med Genet. 2019;56:850–4. doi: 10.1136/jmedgenet-2018-105639 30478137

[26] Pippin JA, Chesi A, Wagley Y, Su C, Pahl MC, Hodge KM, et al. CRISPR-Cas9–Mediated Genome Editing Confirms EPDR1 as an Effector Gene at the BMD GWAS-Implicated ‘STARD3NL’ Locus. JBMR Plus. 2021;5:e10531. doi: 10.1002/jbm4.10531 34532616 PMC8441377

[27] Chesi A, Wagley Y, Johnson ME, Manduchi E, Su C, Lu S, et al. Genome-scale Capture C promoter interactions implicate effector genes at GWAS loci for bone mineral density. Nat Commun. 2019;10. 30890710 10.1038/s41467-019-09302-x PMC6425012

[28] Gupta M, Cheung C-L, Hsu Y-H, Demissie S, Cupples LA, Kiel DP, et al. Identification of homogeneous genetic architecture of multiple genetically correlated traits by block clustering of genome-wide associations. J Bone Miner Res. 2011;26:1261–71. doi: 10.1002/jbmr.333 21611967 PMC3312758

[29] Rao S, Yang X, Ohshiro K, Zaidi S, Wang Z, Shetty K, et al. β2-spectrin (SPTBN1) as a therapeutic target for diet-induced liver disease and preventing cancer development. Sci Transl Med. 2021;13:eabk2267. 34910547 10.1126/scitranslmed.abk2267 PMC8941321

[30] Chen S, Wu H, Wang Z, Jia M, Guo J, Jin J, et al. Loss of SPTBN1 Suppresses Autophagy Via SETD7-mediated YAP Methylation in Hepatocellular Carcinoma Initiation and Development. Cell Mol Gastroenterol Hepatol. 2022;13:949–973.e7. doi: 10.1016/j.jcmgh.2021.10.012 34737104 PMC8864474

[31] Kawao N, Kaji H. Interactions Between Muscle Tissues and Bone Metabolism. J Cell Biochem. 2015;116:687–95. doi: 10.1002/jcb.25040 25521430

[32] Sun L, Tan L-J, Lei S-F, Chen X-D, Li X, Pan R, et al. Bivariate Genome-Wide Association Analyses of Femoral Neck Bone Geometry and Appendicular Lean Mass. PLOS ONE. 2011;6:e27325. doi: 10.1371/journal.pone.0027325 22087292 PMC3210160

[33] Li S, Liu T, Li K, Bai X, Xi K, Chai X, et al. Spectrins and human diseases. Transl Res. 2022;243:78–88. doi: 10.1016/j.trsl.2021.12.009 34979321

[34] Unudurthi SD, Greer-Short A, Patel N, Nassal D, Hund TJ. Spectrin-based pathways underlying electrical and mechanical dysfunction in cardiac disease. Expert Rev Cardiovasc Ther. 2018;16:59–65. doi: 10.1080/14779072.2018.1418664 29257730 PMC6064643

[35] Chen Y-C, Guo Y-F, He H, Lin X, Wang X-F, Zhou R, et al. Integrative Analysis of Genomics and Transcriptome Data to Identify Potential Functional Genes of BMDs in Females. J Bone Miner Res. 2016;31:1041–9. doi: 10.1002/jbmr.2781 26748680

[36] Calabrese GM, Mesner LD, Stains JP, Tommasini SM, Horowitz MC, Rosen CJ, et al. Integrating GWAS and Co-expression Network Data Identifies Bone Mineral Density Genes SPTBN1 and MARK3 and an Osteoblast Functional Module. Cell Syst. 2017;4:46–59.e4. doi: 10.1016/j.cels.2016.10.014 27866947 PMC5269473

[37] Xu X, Yang J, Ye Y, Chen G, Zhang Y, Wu H, et al. SPTBN1 Prevents Primary Osteoporosis by Modulating Osteoblasts Proliferation and Differentiation and Blood Vessels Formation in Bone. Front Cell Dev Biol [Internet]. 2021 [cited 2023 Apr 23];9. Available from: https://www.frontiersin.org/articles/10.3389/fcell.2021.653724 33816505 10.3389/fcell.2021.653724 PMC8017174

[38] Zhi X, Lin L, Yang S, Bhuvaneshwar K, Wang H, Gusev Y, et al. βII‐Spectrin (SPTBN1) suppresses progression of hepatocellular carcinoma and Wnt signaling by regulation of Wnt inhibitor kallistatin. Hepatology. 2015;61:598. 25307947 10.1002/hep.27558 PMC4327990

[39] Ehrlich M. DNA hypermethylation in disease: mechanisms and clinical relevance. Epigenetics. 2019;14:1141–63. doi: 10.1080/15592294.2019.1638701 31284823 PMC6791695

[40] Duffy Á, Verbanck M, Dobbyn A, Won H-H, Rein JL, Forrest IS, et al. Tissue-specific genetic features inform prediction of drug side effects in clinical trials. Sci Adv. 2020;6:eabb6242. doi: 10.1126/sciadv.abb6242 32917698 PMC11206454

[41] Sonawane AR, Platig J, Fagny M, Chen C-Y, Paulson JN, Lopes-Ramos CM, et al. Understanding Tissue-Specific Gene Regulation. Cell Rep. 2017;21:1077–88. doi: 10.1016/j.celrep.2017.10.001 29069589 PMC5828531

[42] Schierding W, Cutfield W, O’Sullivan J. The missing story behind Genome Wide Association Studies: single nucleotide polymorphisms in gene deserts have a story to tell. Front Genet [Internet]. 2014 [cited 2022 Dec 8];5. Available from: https://www.frontiersin.org/articles/10.3389/fgene.2014.00039 10.3389/fgene.2014.00039 PMC3927098 24600475

