# Supplementary material for: Identification of bone mineral density associated genes with shared genetic architectures across multiple tissues: Functional insights for EPDR1, PKDCC, and SPTBN1
Source: PLoS One. 2024 Apr 29;19(4):e0300535. doi: 10.1371/journal.pone.0300535 (PMC11057974; doi:10.1371/journal.pone.0300535)

S1 Fig. The summary per genomic risk locus. Note that genomic loci could contain more than one independent lead Single-Nucleotide Polymorphisms (SNPs).

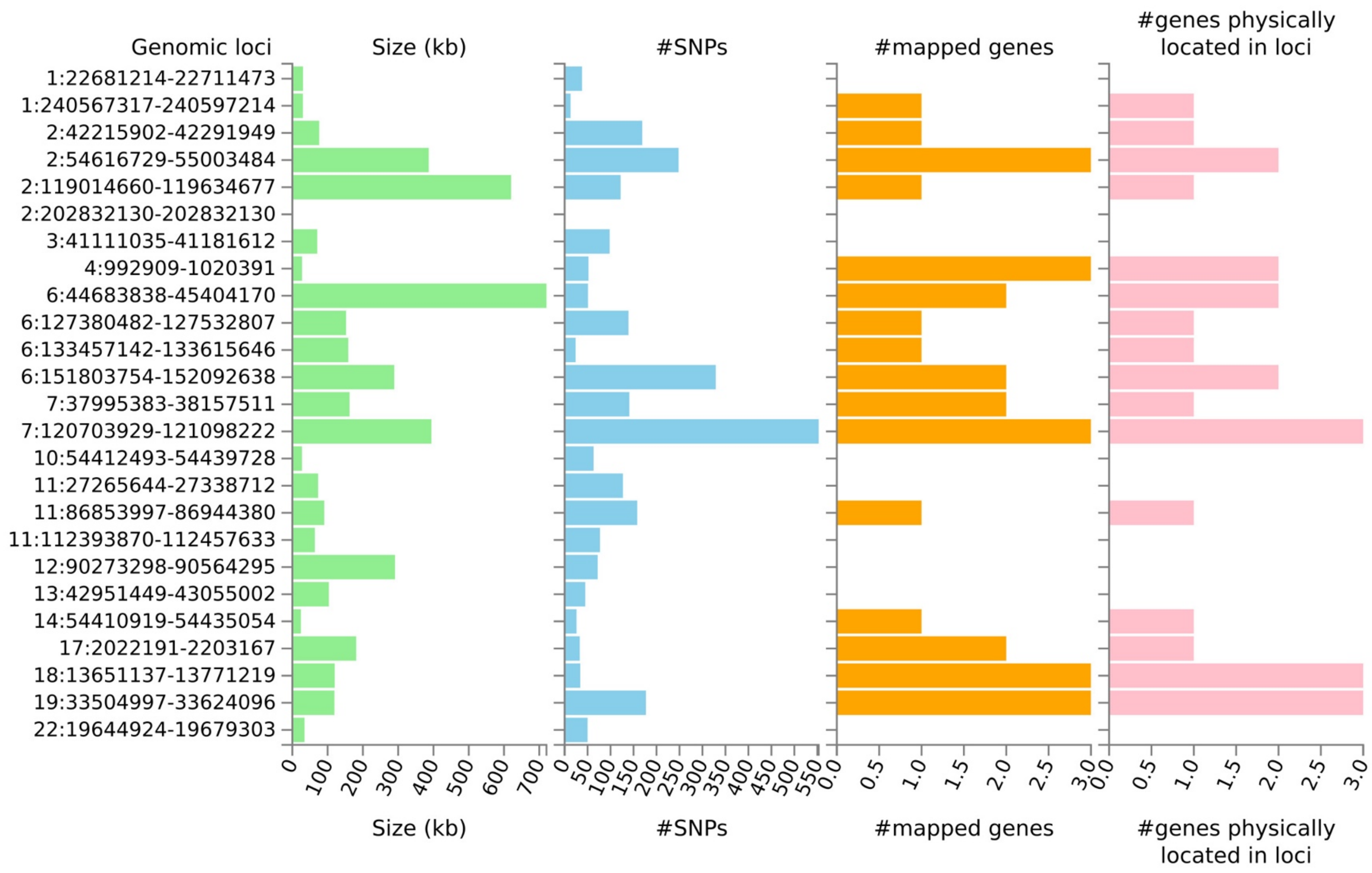

Supplement: S1 Fig — Note that genomic loci could contain more than one independent lead Single-Nucleotide Polymorphisms (SNPs). (PDF) [file pone.0300535.s001.pdf]
