# Supplementary material for: Identification of bone mineral density associated genes with shared genetic architectures across multiple tissues: Functional insights for EPDR1, PKDCC, and SPTBN1
Source: PLoS One. 2024 Apr 29;19(4):e0300535. doi: 10.1371/journal.pone.0300535 (PMC11057974; doi:10.1371/journal.pone.0300535)

S2 Fig. **Tissue enrichment analysis using MAGMA** [20]. The most enriched tissue is the fallopian tube. Significantly enriched DEG sets ($P_{bon} < 0.05$) are highlighted in red. A threshold $P \leq 5 \times 10^{-8}$ was used to map the genes. The horizontal dashed line indicates a Bonferroni-corrected significance threshold with 53 tissue types (0.05/53).

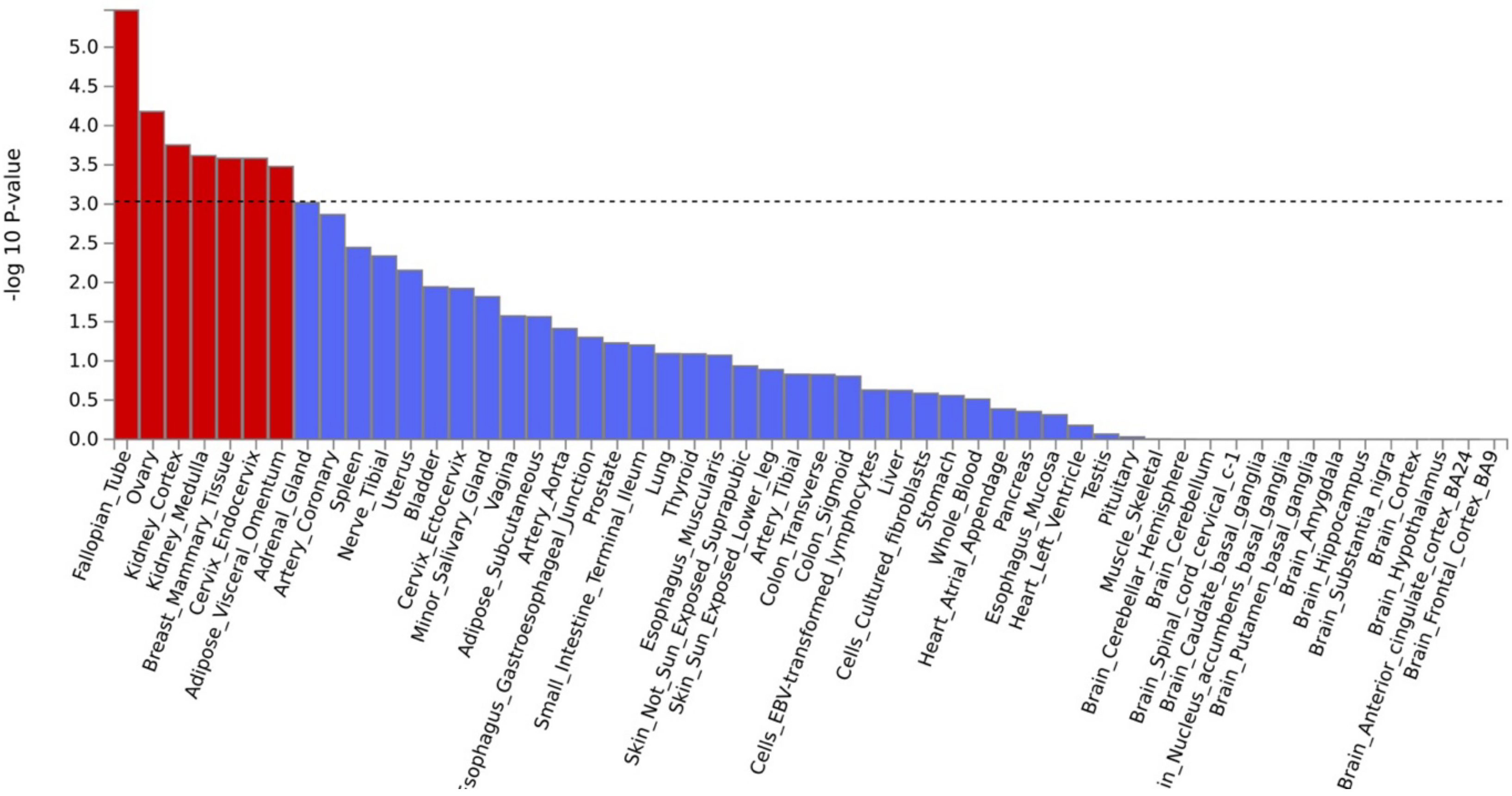

Supplement: S2 Fig — The most enriched tissue is the fallopian tube. Significantly enriched DEG sets (Pbon<0.05) are highlighted in red. A threshold P ≤5 ×10^−8^ was used to map the genes. The horizontal dashed line indicates a Bonferroni-corrected significance threshold with 53 tissue types (0.05/53). (PDF) [file pone.0300535.s002.pdf]
