# Supplementary material for: Identification of bone mineral density associated genes with shared genetic architectures across multiple tissues: Functional insights for EPDR1, PKDCC, and SPTBN1
Source: PLoS One. 2024 Apr 29;19(4):e0300535. doi: 10.1371/journal.pone.0300535 (PMC11057974; doi:10.1371/journal.pone.0300535)

S3 Fig. **Differentially expressed genes (DEG) in 30 major tissues in the GTEx data**. A threshold $P \leq 5 \times 10^{-100}$ was used to map the genes.

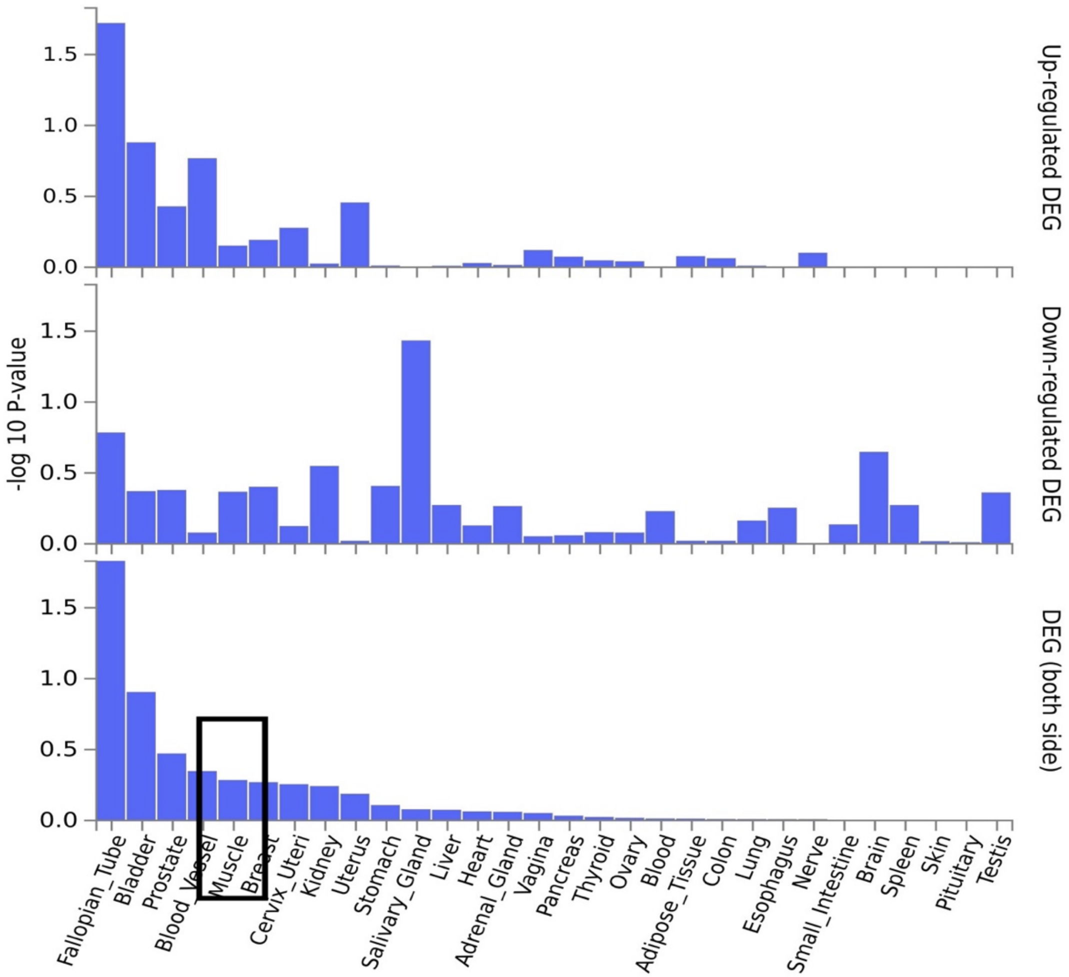

Supplement: S3 Fig — A threshold P ≤5 ×10−100 was used to map the genes. (PDF) [file pone.0300535.s003.pdf]
